# Biomarkers in Different Asthma Phenotypes

**DOI:** 10.3390/genes12060801

**Published:** 2021-05-25

**Authors:** Sanja Popović-Grle, Anamarija Štajduhar, Marina Lampalo, Dina Rnjak

**Affiliations:** 1 The Clinic for Lung Diseases Jordanovac, University Hospital Centre Zagreb, Jordanovac 104, 10 000 Zagreb, Croatia; sanja.popovic.grle@kbc-zagreb.hr (S.P.-G.); marina.lampalo@gmail.com (M.L.); dina.rnjak@gmail.com (D.R.); 2 School of Medicine, University of Zagreb, Šalata 2b, 10 000 Zagreb, Croatia

**Keywords:** asthma phenotype, clusters, biomarkers, eosinophils

## Abstract

Asthma is the most common respiratory disease. It has multiple phenotypes thatcan be partially differentiated by measuring the disease’s specific characteristics—biomarkers. The pathogenetic mechanisms are complex, and it is still a challenge to choose suitable biomarkers to adequately stratify patients, which became especially important with the introduction of biologicals in asthma treatment. Usage of biomarkers and an understanding of the underlying pathobiological mechanisms lead to the definition of endotypes. Asthma can be broadly divided into two endotypes, T2-high and T2-low. The right combination of various biomarkers in different phenotypes is under investigation, hoping to help researchers and clinicians in better disease evaluation since theindividual approach and personalized medicine are imperative. Multiple biomarkers are superior to a single biomarker.

## 1. Introduction

Asthma is the most common chronic respiratory disease and affects both children and adults [1,2]. In 2016, the Global initiative for asthma (GINA) has stated that asthma is a heterogeneous disease, with multiple phenotypes [3]. Phenotype is by definition an observable disease characteristic that is the result of gene–environment interaction. Asthma phenotype description dates from the mid-20th century, with Rackeman’s idea from 1947 about extrinsic and intrinsic asthma emphasizing the triggering role of allergens in asthma [4]. The theme is scientifically attractive even nowadays since there are 8767 articles about asthma phenotypes (accessed in February 2021). Asthma phenotyping is needed for a more accurate patient approach and a better understanding of asthma diversity [5]. Asthma heterogeneity can be seen in diverse clinical presentations, different responses to treatment, different pathophysiological features, and findings due to various pathogenic mechanisms, which lead to multiple asthma phenotypes [5,6]. Asthma phenotypes comprise clusters of patients characterized by similar clinical or biological features [5,6]. Early-onset asthma is typically of allergic phenotypes and has been so far the most extensively investigated. The prevalence of adult-onset asthma is increasing because of the population aging. This asthma phenotype can be divided into two main types considering the existence of eosinophilic inflammation or allergic sensitization. Various other asthma phenotypes, for instance, exercise-induced, and obesity- or smoking-associated asthma, should be taken into account when evaluating the patient. Severe asthma, with the prevalence of 5–10% of all asthma patients, remains a clinical challenge [5,6]. The parameters that help to differentiate asthma phenotypes can be:

- disease symptoms (more cough, wheezing or dyspnea)
- triggers
- body shape and weight
- age of asthma onset
- etiology
- atopic status
- smoking habit
- exposure to professional irritants
- laboratory findings
- biomarkers
- lung function parameters and presence of fixed airflow obstruction
- bronchodilator reversibility
- value of fractional exhaled nitric oxide (FeNO)
- reactions to drugs or substances, especially to aspirin and nonsteroidal anti-inflammatory drugs
- response to therapy, like steroids or anticholinergics, etc.
- level of asthma control
- number of exacerbations
- level of severity, and speed of onset of asthma deterioration
- need for hospitalization or intensive care unit treatment (including mechanical ventilation)
- duration of asthma remission
- the involvement of other organ systems like skin (urticaria/eczema, atopic dermatitis), or digestive system (eosinophilic esophagitis, etc.)
- the involvement of upper respiratory airways and/or nasal polyps, etc. [5,6].

You can see the Asthma phenotyping—evolution and modern approach in Table 1.

The topic concerning asthma phenotypes is very important as personalized, individually tailored treatment adjusted to the understanding of the underlying phenotypic mechanisms yields much better results in asthma patients [5,6].

Only observable characteristics are not enough, because of many overlapping symptoms of the disease, so the common underlying pathophysiological mechanism in asthma is important. Due to these facts, a new term was developed—asthma endotypes, which are defined by specific pathobiological mechanisms at the molecular and cellular levels [7]. While phenotypes are mostly explained and divided through observable (“visible”) characteristics, endotypes are divided using biomarkers, special disease characteristics which could be measured, quantifiable factors that can distinguish between physiological and pathological processes and can be used as a pathway for therapy selection and therapeutic response monitoring [8]. The mechanisms leading to the disease are complex and it is still a challenge to choose suitable biomarkers to adequately stratify patients, which became especially important with the introduction of biologicals in asthma treatment [5]. The key point is biomarkers for the endotypic (and phenotypic) criteria. The right combination of various biomarkers in different endotypes is under investigation, hoping to help researchers and clinicians in better disease evaluation since the individual approach and personalized medicine are imperative [5,6]. Today, defining a severe asthma endotype is a process based on a biomarker-driven approach [8]. There is no perfect biomarker, and unlike for some other disorders (for example, glycated hemoglobin, HbA1c, in diabetes), biomarkers for asthma are less precise, and still not completely known. With (yet) no perfect biomarkers, there is no perfect endotype classification. Some authors suggest division according to sputum cytology, others use clinical and molecular entities to describe asthma endotypes, but most authors agree that, based on biomarkers, asthma can be divided into two groups, T2-high asthma, and T2-low (or non T2) asthma [5,8]. The T2-high and T2-low asthma classification is most commonly used since this is most useful in treatment selection—the available severe asthma targeted therapy is directed to the T2-high molecular pathway. 

## 2. T2-High Asthma Biomarkers

Pathophysiological and inflammatory processes in T2-high asthma are represented by a high level of type 2 cytokines: IL-5, IL-4, IL-13, IL-25, IL-33, and thymic stromal lymphopoietin (TSLP). The most important cells in this type of asthma are Th2 helper CD4+ cells, which secrete these cytokines and activate other innate and adaptive immune cells; basophils, mast cells, B cells [9,10]. In the airways, type 2 innate lymphoid cells (ILC2s) are particularly important in generating type 2 inflammatory responses by producing IL-5 and IL-13 [9,10]. IL-5, IL-3, IL-4, IL-9, and IL-13 are the most important eosinophilic cytokines and work as stimulators of eosinophilic production, bone marrow extrusion, proliferation, and differentiation factors [9,10]. These are engaged in eosinophil maturation in the bloodstream and recruitment and activation in the lungs. IL-25, IL-33, and TSLP are “alarmin” cytokines, involved in eosinophil activation, but also airway hyperresponsiveness and remodeling, Table 2. All these activated cells produce inflammatory molecules that perpetuate and expand the inflammatory process in the airways, causing severe bronchospasm [10].

Clinically, T2-high inflammation is correlated with atopic (allergic) disease and a high level of eosinophils in blood and airways. Nowadays, available biological asthma therapy is reserved for T2-high asthma; it targets allergy molecules IgE and eosinophilic interleukins (IL-5, IL-4, IL-13, TSLP, Table 3). T2 pathological markers are the best-known asthma biomarkers; however, further research is needed in this area to provide better and more detailed diagnosis, and targeted and more powerful treatment, and personalized approach to the patients. Measuring cytokines in blood samples (serum, exhaled breath, sputum, urine) could enhance the current diagnostic algorithm and enable a more thorough patient’s disease profile and personalized approach to therapy selection.

### 2.1. Omics

As mentioned above, phenotypes are the product of gene and environment interplay and these processes can be expressed as measurable markers—metabolomes. The genome, transcriptomic, and proteomic asthma studies started recently and are still ongoing. So far, six T2 gene expression markers have been known: alkaline phosphatase, tissue-nonspecific isozyme (ALPL), Charcot–Leyden crystal protein (CLC), carboxypeptidase A3 (CPA3), chemokine receptor 2 (CXCR2), and deoxyribonuclease l-like 3 (DNASElL3). Expression of these genes is associated with better response to inhaled corticosteroids and can help in distinguishing asthma endotypes [11]. Ongoing proteomics studies of bronchoalveolar lavage identified new molecular factors (IFN-γ, PDGF_BB_, Fit3L, IL-2, TNF-β, CCL27, CXCL7, CTAP-III, GM.CSF, HPLN1, trypsin2, cathepsin G, ARSB, PSA2, etc.) that are elevated in severe asthma patients, compared to those with mild or moderate asthma. Measuring these inflammatory cytokines in airways could verify pathological pathways and possibly detect new possible therapy targets [11].

#### miRNA

In the past few years, with the advent of genome-wide association studies (GWAS) and the detection of pathways and genes of interest in asthma (for example, PGD2-CRTH2, ORMDL3, PI3K/AKT, IL-4-IL-13-JAK-STAT-MAP kinases, adiponectin-iNOS-NF-κB, PGD2-CRTH2, IFNs-RIG, FOXC1-miR-PI3K/AKT) small RNAs that are involved in the gene regulation (microRNAs—miRNAs) have been the topic of research [12,13,14,15,16]. The miRNA-mediated gene expression modification starts with the transcription of introns into primary miRNAs (pri-miRNAs), which are then further processed into precursor miRNAs (pre-miRNAs) and mature miRNAs. Mature miRNAs are then capable of mRNA interactions with variable effects on cellular function [17]. Several miRNAs (for instance, miR-21, miR-135a, miR-142, miR-143, miR-146b, miR-193b and miR-223, miR-365, miR-375, miR-452, miR-1165-3p) are common to different diseases with pronounced Th2 activity, which was proven in both humans and murine models [17,18,19,20,21]. Apart from that, miRNAs can also be used as a discriminatory factor for the diagnosis of asthma. One of the most common challenges in clinical practice is distinguishing asthma from chronic obstructive pulmonary disease (COPD). This can be difficult in the case of severe asthma when permanent airway remodeling is present. In the paper written by Lacedonia et al. it was shown that miRNA-145 and miRNA-338 obtained from sputum could be used to differentiate patients with asthma from those with COPD or asthma–COPD overlap [22]. Another potential use of miRNAs is in determining disease activity. Sometimes the presence of asthma remission is not easily established and in part relies on the information accuracy obtained from patients. With the measurement of miRNA expression levels, it is possible to detect persistent asthma even in the absence of other clinical data [23]. Interestingly, miRNA profile could also be utilized to distinguish asthma in remission from completely healthy persons (a problem that sometimes appears in the case of the first-ever visit to the pulmonologist), to predict patients with exacerbation-prone disease and those who will favorably respond to corticosteroids as well as other frequently used medications [23,24,25,26]. Ultimately, the common goal of all these research studies is to understand pathways leading to the onset of asthma and to create novel therapeutic modalities as has already been proven to be possible in animal models [27].

### 2.2. Blood Biomarkers

#### 2.2.1. Blood Eosinophils and Markers of Eosinophil Activation

Eosinophils are specialized leukocytes, primarily found in tissues, and in the respiratory system, airway mucosa, and airways. The exact correlation between blood and airway eosinophils is incompletely understood. Blood eosinophils are easily obtained, but their levels depend on the time of sampling (highest at midnight, lowest at midday), time since eating, exercise, and therapy (corticosteroids reduce eosinophilia) [11]. Eosinophilia is not a diagnostic criterion for asthma but can be found in patients with early-onset allergic and late-onset non-allergic asthma. Moreover, blood eosinophils are asthma prognostic criteria and markers for biological therapy induction. The exact cut-off value to define high blood eosinophil count is still debatable, but most authors suggest a range above 150–300 cells/μL. Some studies showed that patients with eosinophil count above 300 cells/μL have more frequent exacerbations and acute respiratory events [11].

Eosinophils promote inflammation by releasing an abundance of inflammatory mediators that, together with mediators released from T2 cells, cause eosinophilic inflammation, bronchoconstriction, and airway remodeling [28,29,30]. The predominant mediators in the eosinophil granules are cytotoxic cationic proteins, such as eosinophil cationic protein (ECP), eosinophil-derived neurotoxin (EDN), major basic protein (MBP), and reactive oxygen species (ROS) [28,29,30]. Some of these mediators can be measured in blood and used as a guide in better asthma clustering, but also as a potential treatment target. High ECP levels are detected in blood and sputum of severe asthma patients (mostly atopic), compared to those with non-severe asthma [28,29,30]. ECP is associated with bronchospasm and airway resistance and is elevated in asthma exacerbation, and its levels are reduced after therapy induction. It is assumed that ECP can be used as a marker for corticosteroid induction and dosage, but this needs to be confirmed [8]. EDN is another marker of eosinophilic disease and persistent airflow limitation in severe asthma patients; it can be measured in serum, urine, and other body fluids. In childhood asthma, it is a marker of differentiation of stable asthma and asthma exacerbation [31].

#### 2.2.2. Periostin

Evidence shows that a matricellular protein, periostin, is involved in many basic biological processes such as cell proliferation, invasion, and angiogenesis [32]. In recent decades, the number of periostin studies has been rapidly growing, probably due to the wide range of functions of periostin. The protein also takes part in complex pathophysiological mechanisms involved in inflammatory processes and eosinophil recruitment in asthma, airway remodeling, and the development of a T2 phenotype [32]. Periostin has been localized in deposits of subepithelial fibrous tissue in asthmatic patients, and periostin levels have been linked to increases in IL-13 [32]. A murine model study conducted by Bentley et al. found that periostin plays a crucial role in airway hyperresponsiveness and T-cell mediated inflammation after house dust mite sensitization [33]. In a recent study conducted by Takahashi et al. one of the findings was that serum periostin levels were good predictors of blood eosinophilia (*r* = 0.36), which could also mean that periostin levels serve as a biomarker of eosinophilic airway inflammation. Additionally, serum periostin levels proved superior in predicting fixed airflow limitation (FEV1/FVC ratio < 70%) compared to other biomarkers of eosinophil inflammation in asthmatics. Multivariate analysis showed that high serum periostin levels (≥97 ng/mL) positively correlated with fixed airflow limitation (odds ratio 3.2) [34]. Furthermore, this is in agreement with the Biomarkers in Corticosteroid-refractory Asthma (BOBCAT) study, which found an increase in serum periostin levels in asthmatic patients with eosinophilic airway inflammation [35]. In contrast, an earlier study by Agacheet al. found that periostin serum levels did not correlate with any of the type 2 biomarkers, and even after stratification, periostin failed to identify blood or sputum eosinophilia [36].

In another in vitro study, IL-13 caused periostin (POSTN), chloride channel regulator 1 (CLCA1), and serpin peptidase inhibitor clade B member 2 (SERPINB2) gene induction in the lung epithelial cells in comparison with healthy control subjects and chronic obstructive disease patients [36]. Thus, epithelial expression of POSTN, CLCA1, and SERPINB2 genes could be an alternative T2 biomarker [37]. 

In the study conducted by Tajiri et al. the decrement of periostin and serum IgE levels positively correlated with the reduction in exacerbations during the two years. Because of this, it is possible that baseline serum periostin levels could be useful in evaluating response to omalizumab treatment [38]. The special problem with periostin is its unavailability for clinical and routine testing.

Periostin can be used as guidance for anti-IL-13 biological therapy induction since the results ofongoing phase 2 clinical trials showed that patients with high periostin levels have more significant exacerbation reduction and pulmonary function improvement, but according to other studies, usage of periostin as a therapeutic marker may be limited [29,30]. Values above 50 ng/L are considered high periostin levels in most studies [29,30]. The limitation of periostin is that it is also secreted by osteoblasts and the levels can be elevated in some tumors (brain tumors, bony metastasis), and growing children [29].

#### 2.2.3. Dipeptidyl Peptidase-4

Dipeptidyl peptidase-4 (DPP-4) is expressed in different lung cells, but its role in human asthma is uncertain. In rat models, DPP-4 can be found in bronchoalveolar lavage (BAL) and correlates with airway inflammation [10,30]. Studies of DPP-4 in humans are limited; it is assumed that IL-13 stimulates DPP-4 production and, like periostin, DPP-4 can be measured in serum and may be used as a guide for anti-IL-13 therapy induction [10,30]. Brightling et al. reported that high DPP-4 levels correlate with response to anti-IL-13 therapy. On the contrary, other studies did not find a connection between DPP-4 and other asthma markers [10,30].

#### 2.2.4. Osteopontin

Osteopontin is a polyfunctional cytokine produced by several immune cells and epithelial cells. It can be found in Th1-mediated pulmonary disorders, such as pulmonary fibrosis and granulomatous diseases, but recently its role in T2 inflammatory, allergic, response has been observed [29,39]. It has an important role in eosinophil migration and mast cell activation in the airways and can be detected in serum and respiratory samples of asthma patients, compared with healthy people [39]. The results on the usage of osteopontin to estimate asthma severity are contradictory. Further research is needed [29].

#### 2.2.5. Immunoglobulin E (IgE)

Another useful biomarker in patients suffering from asthma is IgE. It is a product of B lymphocytes in reaction to foreign antigens [40]. The total IgE concentration varies depending on the age and numerous extrinsic and intrinsic factors, including IL-4 and -5 [40,41]. During the severe exacerbations, the total IgE level rises, then falls with the resolution of exacerbation, and is expected to achieve relatively stable levels within 1–1.5 months after the beginning of a severe exacerbation [42]. In unselected adult-onset asthma patients the diagnostic utility of total IgE, as a single biomarker, is poorer compared to either fractional exhaled nitric oxide (FeNO) or blood eosinophils, while the combination of FeNO and blood eosinophils further enhances the diagnostic efficacy regardless of asthma phenotype [43]. The total IgE is also less accurate in atopic and obese patients than in non-atopic and non-obese patients, which could be explained by the complex interaction of different sensitizations producing a single allergic phenotype [44]. Ultimately, various specific immunoglobulins E and their interactions could be an important causative mechanism in the development of asthma [44]. Despite this, with the advent of targeted asthma therapy, it has gained a crucial role in individual therapy tailoring as well as in the monitoring of its efficacy. Depending on the blood levels, the total IgE can also point to other associated comorbidities, such as allergic bronchopulmonary aspergillosis even in the presence of corticosteroids [45], which can be useful in severe asthma management.

### 2.3. Respiratory Biomarkers

Studies have demonstrated the benefit of induced sputum to guide asthma treatment and have shown that normalizing airway eosinophilic inflammation correlates with better asthma control with reduced numbers of exacerbations and hospital admissions [46]. The technique of induced sputum that allows non-invasive collection of airway cells is considered the gold standard to identify inflammatory asthma phenotype [47]. One study showed that, compared to the paucigranulocytic phenotype, eosinophilic, neutrophilic, and mixed granulocytic phenotypes were characterized by a poorer lung function. Eosinophilic phenotype exhibited a higher frequency of atopy, higher levels of IgE, higher bronchial hyperresponsiveness to methacholine, higher FeNO levels, and lower asthma control compared to paucigranulocytic [48]. Independent predictors of high sputum eosinophil count (≥3%) were the percentage of blood eosinophils, low FEV1/FVC, high FENO, and IgE levels. A cut-off value of 220/mm^3^ or 3% for blood eosinophils performed equally to FeNO 50 ppb to identify the presence of a sputum eosinophil count ≥3% [47]. Sputum eosinophils >3% can distinguish between eosinophilic vs. neutrophilic, mixed, or paucigranulocytic asthma phenotype [49]. Using blood eosinophilia per se as a biomarker for eosinophilic asthma is difficult due to the daily fluctuations of blood eosinophils, with or without treatment [36]. In the study byAgache et al. serum, IL-5, and IL-13 were identified as the best blood eosinophilia predictors, with a good reproducibility at repeated testing after 6 weeks [36]. 

Sputum cytokines are being researched as potential biomarkers. Eosinophil peroxidase (EPX) is released from activated eosinophils, and can be used as a diagnostic and dynamic asthma marker, since its levels are elevated in sputum, nasal, and pharyngeal swabs of patients with uncontrolled asthma and are reduced after anti-eosinophilic therapy [31]. 

#### 2.3.1. Exhaled Breath Analysis

Around 10% of patients fail to provide adequate samples for induced sputum to be adequately performed [50]. Exhaled breath condensate (EBC) analysis is an easy, non-invasive method of sampling airway material. It enables analysis of volatile and nonvolatile particles, including cytokines, leukotrienes, nitric oxide products, and hydrogen peroxide [51]. The hydrogen peroxide, nitrate, and 8-isoprostane levels are elevated and pH is reduced in asthma patients and can correlate with poor asthma control and the severity of the disease [51]. These are reduced after corticosteroid therapy induction [51]. The gas chromatography and electronic nose (e-nose) detect volatile organic compounds (VOCs) in the exhaled gas and generate a specific “breath-print” for each individual [52]. Prior studies have provided evidence supporting the use of the e-nose to aid in the diagnosis of asthma by discriminating asthmatics from healthy controls or chronic obstructive pulmonary disease (COPD) patients [52]. The exhaled gas contains a complex mix of VOCs derived from various metabolic and inflammatory pathways in the lung. Various diseases, including asthma, are associated with specific breath-prints that can be detected with an e-nose [53]. In the research conducted by Plaza et al. results indicated that e-nose VOC analysis can reliably discriminate airway inflammatory phenotypes in asthma patients with similar clinical manifestations, as was also shown in another paper where gas chromatography–mass spectrometry was used to distinguish eosinophilic from non-eosinophilic asthma phenotypes as well as neutrophilic from non-neutrophilic phenotypes with great accuracy [53], even better than could be done using sputum eosinophil counts or FeNO measurements [54].

Temperature can be measured in exhaled breath since heat is one of the inflammation signs. Asthma patients have higher exhaled breath temperature (EBT) compared with the healthy subjects; research on cut-off values is ongoing [51]. 

According to experts’ opinion, exhaled breath analysis is one of the most important future asthma biomarkers. Unfortunately, it is still used only within studies [55].

#### 2.3.2. Fractional Exhaled Nitric Oxide (FeNO)

FeNO has a distinctive position between all biomarkers because of its importance in the differential diagnostic process, and also as a predictor of different asthma phenotypes, and a biomarker that could predict treatment response [56]. 

Nitric oxide (NO) is a gas normally found in exhaled breath in all humans. Patients with asthma often exhibit higher inducible nitric oxide synthase (iNOS2) levels, the enzyme in charge of epithelial NO production [57].

Different technologies for the measurement of exhaled NO are available—chemiluminescence instruments, electrochemical devices, and laser-based sensors, each one with its advantages and disadvantages. However, electrochemical devices are portable, rather cheap, and offer good precision and reproducibility and, that way, have the potential to be readily utilized even at the point of care [58].

FeNO has a large potential in clinical practice due to easy measurement, the non-invasive nature of the test, and the ease of repeat measurements. It provides additional insight into the status of airway inflammation, by adding a new dimension to well-established clinical tools—history, physical exam, and lung function tests. Many factors affect FeNO values and there is considerable overlap between mean FeNO values in healthy individuals and those with stable asthma. The American Thoracic Society recommends clinically significant cut-off points for FeNO: (1) <25 ppb (<20 ppb in children), and (2) >50 ppb (>35 ppb in children) [59].

FeNO is a biomarker of T2 response (or airway eosinophilia) but does not correlate with sputum eosinophils. A FeNO level >50 ppb indicates a probablygood steroid response, while patients with a FeNO level around 25 ppb are less likely to respond to steroids [59].

A recent study by Price et al. found that people with a combination of high FeNO and high blood eosinophils were prone to a notably increased risk of severe exacerbations in the year preceding FeNO measurement. For instance, patients with a high FeNO (≥50 ppb) and high blood eosinophil count (≥0.300 × 10^9^ cells/L) were almost four times as likely to have a severe exacerbation as patients with non-high FeNO (<25 ppb) and non-high blood eosinophils preceding the FeNO reading [60]. Studies showed conflicting results on the diagnostic accuracy of blood eosinophils or FeNO between mildtomoderate and severe asthma patients [61]. 

Neelageman et al. found that there is no significant difference in the observed baseline FeNO values between different groups of patients, categorized according to asthma severity and ICS response (mild vs. moderate vs. moderately severe persistent asthmatics; ICS good vs. poor responders). However, there was a statistical difference in baseline and post-treatment FeNO levels in those groups depending on the ICS response. This study does not support the predictive power of baseline FeNO values in future ICS response and asthma severity but FeNO level reduction is related to clinical improvement with no implication on respiratory function [61]. 

The diagnostic accuracy of FeNO and blood eosinophils to detect sputum eosinophilia does not significantly differ between obese and non-obese, atopic and non-atopic, (ex-)smoking and never-smoking, and severe and mild-to-moderate asthma patients [43]. The aim of the study conducted by Bernholm et al. was to assess FeNO versus symptom-guided treatment tailoring in an attempt to gain asthma control. This double-blinded, parallel, randomized control trial included 80 patients with asthma, and the treatment was tailored using either a FeNO or Asthma Control Questionnaire-based algorithm. In the FeNO-guided group, airway hyperresponsiveness showed improvement (*p* = 0.015); however, after 36 weeks, groups showed no disparity (*p* = 0.3) [62]. 

A recent study by Brooks et al. found that the implementation of FeNO into pre-omalizumab treatment assessment decreases the expected per-patient cost by almost 50% from the moment of omalizumab initiation into therapy, and the same trend continues during the first year of the omalizumab treatment. The authors point to the obvious benefit of using FeNO for detecting omalizumab responders (before initiating a 12-week trial of omalizumab) [63,64].

### 2.4. Urine Biomarkers

#### 2.4.1. Bromotyrosine

Bromotyrosine is a metabolite of tyrosine protein produced by activated lung eosinophils. It is suggested that its concentration is elevated in allergic asthma patients and may correlate with the severity of airflow limitation and maybe as a prognostic factor for future exacerbations, Table 2. Concordance with other biomarkers (sputum eosinophils, FeNO) is not high [8].

#### 2.4.2. Prostaglandin D2 and Leukotriene E4

Prostaglandin D2 (its urine metabolite 9α,11β-PGF2) and leukotriene E4 may be used as aspirin-exacerbated respiratory disease (AERD) markers. AERD is characterized by the combination of severe asthma, recurrent nasal polyposis, and the occurrence of acute respiratory symptoms after exposure to non-steroidal anti-inflammatory drugs (NSAIDs). The pathological mechanism involves arachidonic acid metabolism with dysregulation of PGD2 and PGE2 levels and up-regulation of leukotriene levels. Levels of these cytokines are elevated in urine post-exposure to NSAID and correlate with the severity of bronchospasm [31,51].

## 3. T2-Low Asthma Biomarkers

Unlike T2-high asthma, there are only a few potentially useful biomarkers that could be used to diagnose and monitor T2-low asthma (mediated by Th1 and Th17 cells) [64].

Lately, a novel biomarker has been introduced. MicroRNAs (miRNAs), small, highly conserved, non-coding RNA molecules which are responsible for the control of genomic expression. A single miRNA can affect multiple genes, but multiple miRNAs can also modify the transcription of a single gene [65]. They affect translation and are involved in cellular processes in many ways [65]. They were, at first, mostly investigated in oncology, but since the discovery of their role in allergic diseases and other immunological disorders, miRNAs have been utilized as a biomarker for T2-low asthma [65]. It was shown that different expressions of various miRNAs in smooth muscle cells, epithelium, and the immune system can discriminate between healthy controls and people suffering from asthma [65]. It was also found that the upregulation of certain miRNAs can result in steroid resistance, rise in the IL-8 in the epithelial cells, and damaged pulmonary function which is the hallmark of neutrophilic asthma, the main non-T2 asthma phenotype [66].

The positive side of miRNAs is that they can be obtained not only from lung tissue biopsies but also from the induced sputum, which is an easy-to-conduct and relatively safe method for obtaining valuable samples.

However, unlike the T2-high asthma biomarkers that can at the same time be targets of the biologic therapies, we are still far from utilizing miRNAs as the specific therapeutic objects due to the uncertainty of the intervention’s results. For instance, blocking miRNAs responsible for non-T2 asthma could potentially have deleterious effects on other cellular pathways and cause dangerous side effects.

Other potential serum biomarkers are cytokines, chemokines, and specific molecules, which are elevated in T2-low asthma, such as IL-1β, IL-6, IL-8, IL-17, brain-derived neutrophilic factor (BDNF), S100A9, folliculin (secreted in case of epithelial damage), myeloperoxidase, neutrophil elastase, and tumor necrosis factor (TNF)α [31,64,67].

These have a confirmed role in airway remodeling, asthma development after an infection or environmental influence, and maintenance of neutrophilic or paucigranulocytic inflammation, and inflammatory cell degranulation [68,69,70]. 

Breath volatile organic compounds, for example, nonanal, 1-propanol, and hexane, were also investigated as neutrophilic asthma indicators [67]. It is worth noting that the levels of these molecules are generally the highest in moderate and severe asthma [71].

Unfortunately, all potential biological drugs against some of these molecules were similarly ineffective in clinical trials [67,72]. 

Additionally, cell surface antibodies could also be of use in asthma phenotype differentiation [73].

Interestingly, previously thought as an exclusively eosinophilic biomarker, ECP was found also to be elevated in neutrophilic asthma in the absence of eosinophils, and osteopontin, which was regarded as a T1-biomarker, was found to be elevated in asthma in general, but could not be used to differentiate between phenotypes, showing that future investigations could change old dogmas [74,75].

## 4. Multiple Biomarkers Are Superior to a Single Biomarker

Asthma is a complex entity and has many underlying mechanisms. Clinicians and researchers have multiple biomarkers at hand each with its respective advantages and limitations. As such, it was hypothesized that multiple biomarkers are crucial for adequate asthma diagnosis [63].

In a study conducted by Mansur et al. it was surprisingly found that there was no benefit from a composite score of the three biomarkers [76] but it may be due to our current lack of knowledge of complete pathophysiological mechanisms in patients with asthma and thus the inability to choose suitable biomarker combinations. Despite that, in studies, it is usually proposed that the panel of biomarkers has positive implications in decision making when creating an asthma therapy plan and there is hope that further understanding of omics in asthma could lead to a satisfactory composite set of biomarkers [77].

Everyday clinical practice requires pragmatism and feasibility to enable accurate and punctual diagnosis and treatment. In this context, the above-mentioned text, and the various biomarkers’ existence, can complicate daily asthma workup and therapy selection. (Un)fortunately, most biomarkers are not routinely used—many of them are not commercially available or are too expensive—and are used only in the context of medical research studies. Therefore, the cost of measurement and the exact utility in everyday work are unknown. The bottom line is that asthma specialists measure IgE, FeNO, and (blood and tissue) eosinophils in clinical practice, since these are used in available targeted biological therapy. Many problems remain in this area: availability of more biomarkers in clinical routine, advanced biomarker combinations to accurately detect biological responsiveness, detection of biomarkers for therapy monitoring, development of T2-low biomarkers, etc.

## Figures and Tables

**Table 1:** Asthma phenotyping—evolution and modern approach.

Asthma phenotypes according to etiology
Allergic asthma—previously extrinsic
Non-allergic asthma—previously intrinsic
Aspirin exacerbated respiratory disease AERD (usually connected to nasal polyposis, Samter’s triad or syndrome de Widal)
Exercise-induced asthma
Occupational asthma
**Asthma phenotypes according to clinical characteristics**
Obesity-related asthma
Smoking-associated asthma
Cough variant asthma
Persistent asthma
Intermittent asthma
Premenstrual asthma
Preschool asthma
Post-puberty asthma
Early-onset asthma
Infantile asthma
Late-onset asthma
Very late-onset asthma
Exacerbations-prone asthma
Atypical asthma
Classic asthma
**Asthma phenotypes with underlying diseases**
Eosinophilic granulomatosis with polyangiitis (Churg–Strauss syndrome)
Allergic bronchopulmonary mycosis (ABPM)
Asthma with bronchiectasis
Asthma with immunodeficiency
Asthma with α-1 antitrypsin deficiency (AATD)
**Asthma phenotypes according to pulmonary function results**
Reversible asthma (with normalization of lung function)
Asthma with fixed airway obstruction (FAO)
Asthma with non-reversible airflow limitation (negative bronchodilatortest to salbutamol)
Restrictive ventilatory disorders such as asthma
Airway hyperresponsiveness
Asthma with a high inflammatory component (measured by fractional exhaled nitric oxide FeNO)
Asthma with a low inflammatory component (measured by fractional exhaled nitric oxide FeNO)
Brittle asthma (wide variation of peak expiratory flow (PEF))
**Asthma phenotype according to cellular composition of airway inflammation**
Eosinophilic asthma
Neutrophilic asthma
Mixed asthma
Paucigranulocytic asthma
**Asthma phenotypes based on treatment response and level of asthma control**
Severe asthma
Difficult–to-treat asthma
Refractory asthma
Treatment-resistant asthma
Problematic asthma
Uncontrolled asthma
Steroid-resistant asthma
Steroid-dependent asthma
Asthma with a history of respiratory failure and/or intubation and mechanical ventilation
Mild asthma
Benign asthma
**Asthma phenotypes based on the level of type 2 cytokine profile (modern approach)**
T2 high asthma
T2-low (or non T2-high)
**Asthma phenotypes according to etiology**
Allergic asthma—previously extrinsic
Non-allergic asthma—previously intrinsic
Aspirin exacerbated respiratory disease AERD (usually connected to nasal polyposis, Samter’s triad or Syndrome de Widal)
Exercise-induced asthma
Occupational asthma

**Table 2:** T2-high asthma biomarkers.

Examples of T2-High Asthma Biomarkers	
Omics	ALPL, CLC, CPA3, CXCR2, DNASElL3, PGD2-CRTH2, ORMDL3, PI3K/AKT, IL-4-IL-13-JAK-STAT-MAPK, adiponectin-iNOS-NF-κB, PGD2-CRTH2, IFNs-RIG, FOXC1-miR-PI3K/AKT
miRNA	miR-21, miR-135a, miR-142, miR-143, miR-146b, miR-193b and miR-223, miR-365, miR-375, miR-452, miR-1165-3p
Blood biomarkers	Eosinophils, ECP, EDN Periostin DPP-4 Osteopontin IgE
Respiratory biomarkers	Sputum analysis Exhaled breath analysis FeNO
Urine biomarkers	Bromotyrosine PGD2, PGE2, leukotriene E4

**Table 3:** Biological severe asthma therapy.

Biologics for Severe (T2-High) Asthma	
Target	Drug
IL-4 receptor	Dupilumab
IL-5	Mepolizumab Reslizumab
IL-5 receptor	Benralizumab
IgE	Omalizumab

## Data Availability

We choose to exclude this statement because the study did not report any data, all is cited in references.
